# Conventional and microfluidic methods: Design and optimization of lipid-polymeric hybrid nanoparticles for gene therapy

**DOI:** 10.1007/s13346-024-01644-4

**Published:** 2024-06-13

**Authors:** Daniel González-García, Olga Tapia, Carmen Évora, Patricia García-García, Araceli Delgado

**Affiliations:** 1 Department of Chemical Engineering and Pharmaceutical Technology, Universidad de La Laguna, La Laguna, 38200 Spain; 2 Institute of Biomedical Technologies (ITB), Center for Biomedical Research of the Canary Islands (CIBICAN), Universidad de La Laguna, La Laguna, 38200 Spain; 3 Department of Basic Medical Sciences, Universidad de La Laguna, La Laguna, 38200 Spain

**Keywords:** Lipid-polymeric hybrid nanoparticles, Microfluidics, Gene therapy, Nanoparticles stability, Endosomal escape

## Abstract

Gene therapy holds significant promise as a therapeutic approach for addressing a diverse range of diseases through the suppression of overexpressed proteins and the restoration of impaired cell functions. Developing a nanocarrier that can efficiently load and release genetic material into cells remains a challenge. The primary goal of this study is to develop formulations aimed to enhance the therapeutic potential of GapmeRs through technological approaches. To this end, lipid-polymeric hybrid nanoparticles (LPHNPs) with PLGA, DC-cholesterol, and DOPE-mPEG_2000_ were produced by conventional single-step nanoprecipitation (SSN) and microfluidic (MF) methods. The optimized nanoparticles by SSN have a size of 149.9 ± 18.07 nm, a polydispersity index (PdI) of 0.23 ± 0.02, and a zeta potential of (ZP) of 29.34 ± 2.44 mV, while by MF the size was 179.8 ± 6.3, a PdI of 0.24 ± 0.01, and a ZP of 32.25 ± 1.36 mV. Furthermore, LPHNPs prepared with GapmeR-protamine by both methods exhibit a high encapsulation efficiency of approximately 90%. The encapsulated GapmeR is completely released in 24 h. The LPHNP suspensions are stable for up to 6 h in 10% FBS at pH 5.4 and 7.4. By contrast, LPHNPs remain stable in suspension in 4.5% albumin at pH 7.4 for 24 h. Additionally, LPHNPs were successfully freeze-dried using trehalose in the range of 2.5-5% as cryoprotectant The LPHNPs produced by MF and SSN increase, 6 and 12 fold respectively, GapmeR cell uptake, and both of them reduce by 60–70% expression of Tob1 in 48 h.Our study demonstrates the efficacy of the developed LPHNPs as carriers for oligonucleotide delivery, offering valuable insights for their scale up production from a conventional bulk methodology to a high-throughput microfluidic technology.

**Graphical Abstract:**

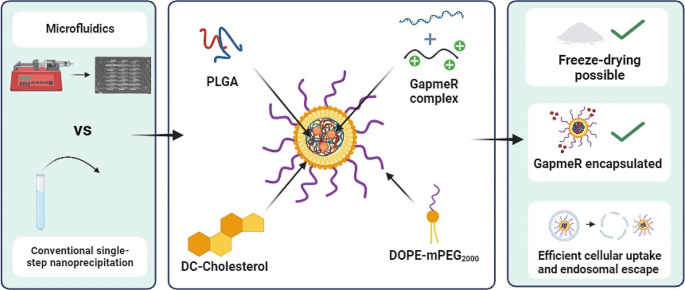

**Supplementary Information:**

The online version contains supplementary material available at 10.1007/s13346-024-01644-4.

## Introduction

Gene therapy is a promising therapeutic option to treat a wide range of diseases by silencing over-producing proteins and genetically modifying and repairing cell functions that may affect disease conditions [1]. In the field of oligonucleotide-based therapies, GapmeRs are an emerging class of molecules that can knock down a target gene through post-transcriptional gene silencing [2]. Despite the great potential of GapmeRs, there are certain biopharmaceutical problems such as degradation risk, difficulties diffusing through biological membranes, and uncontrolled biodistribution due to their lack of specificity, low stability, and poor cellular internalization [3].

To overcome these issues, many formulation advances have been made over the past 30 years, including its encapsulation into non-viral delivery vectors [4]. Due to their minimal cytotoxicity and capacity to offer high oligonucleotide stability in the blood, lipid-polymeric hybrid nanoparticles (LPHNPs) have been shown to be highly successful for drug administration [5]. They comprise two essential components: a polymer core to encapsulate the drug and a lipid monolayer enveloping the core. The polymeric core provides stability and physical integrity during storage. In addition, the lipid shell serves as a molecular blockade, decreasing the leakage of enclosed drugs and shielding the core from deterioration by blocking water infiltration. The lipids also promote cellular uptake and can be functionalized with cellular markers to increase specificity and reduce rapid clearance from blood circulation. These systems combine the advantages of lipid and polymeric nanoparticles (NPs), resulting in enhanced stability, biocompatibility and cellular uptake [5, 6].

However, the industrial development of LPHNPs is slowed by the lack of adequate production technology. Consequently, their clinical implementation progresses slower than conventional drug delivery systems. This is primarily due to the limitations associated with bulk preparation techniques. Single-step nanoprecipitation (SSN) is a widely used method for the preparation of LPHNPs because it is simple, rapid and economical [7]. However, its application by conventional bulk technology leads to challenges related to scaling up production, low reproducibility across batches, and the wide size distribution observed in the resulting NPs [8].

A recent advance in the production of NPs is microfluidic technology (MF). Microfluidics involves the laminar mixing of small volumes of solutions within milliseconds, allowing for the tuning of NPs properties such as size, surface charge, and encapsulation efficiency (EE) by changing concentrations of precursor reagents, the flow rate ratio (FRR), defined as the ratio between the flow rate of aqueous and organic solutions, and the total flow rate (TFR), defined as the sum of aqueous and organic flow rates [9, 10]. Compared to conventional bulk techniques, the MF offers advantages in terms of standardization, certification, reproducibility and the facility to scale up production. Scalability in the production of oligonucleotide nanocarriers at an affordable cost is another important challenge to overcome for their widespread clinical use.

It is important to point out that designing an effective nanocarrier to mitigate the issues associated with GapmeRs is a challenging task. The biological system’s microenvironment, which is meant to be treated, has the ability to alter the properties of the nanocarrier.

First of all, the GapmeRs can be complexed with cationic materials including protamine, DC-cholesterol, and chitosan to increase the yield of oligonucleotide encapsulation and enable their core localization. DC-cholesterol has been widely employed as a transfection agent [11, 12] while low-molecular-weight protamine sulfate can shield oligonucleotides from biological degradation and promote their cellular penetration [13, 14]. Because of its endosomolytic and oligonucleotide complexation capabilities, chitosan has also been used [15].

Size and zeta potential (ZP) must be carefully optimized in the preparation of NPs. Size plays a crucial role in cellular uptake with smaller NPs exhibiting higher uptake than their larger counterparts. Size also affects drug release kinetics and biodistribution. NPs surpassing a diameter of 200 nm will trigger activation of the complement system and subsequently experience swift elimination from the bloodstream, resulting in their accumulation within the liver and spleen [16–18]. ZP determines cellular uptake, biodistribution, and interactions within biological environments [19]. It has been observed that the cationic charge of NPs is strongly correlated with increased cellular uptake but is also associated with greater complement activation compared to negatively or neutrally charged particles [20, 21].

Consequently, the use of ionizable lipids, such as cholesterol derivatives with tertiary amino groups, is an interesting strategy. These lipids are neutral at physiological pH to minimize toxicity and they can become protonated in the acidic endosome pH after their cellular uptake. This protonation facilitates membrane fusion or disruption, endosomal escape, and the release of cargo into the cytosol [22]. The 3-[N-(N, N - dimethylaminoethane) carbamoyl] cholesterol (DC-Chol) offers the advantages of providing greater stability to lipid membranes and lower cytotoxicity as it is a molecule derived from the natural component cholesterol [12]. The utilization of pegylated lipids such as DOPE-mPEG_2000_ (1,2-distearoyl-sn-glycero-3-phosphoethanolamine-N-[methoxy(polyethylene glycol)-2000) also enhances the stability of formulations and prolongs the circulation times of these systems. Despite the characteristics of these lipids, the use of DC-Chol and DOPE-mPEG_2000_ in combination has only been widely used in the preparation of liposomes. In this study, the possible synergistic effect of both lipids on the properties of LPHNPs was explored.

The main objective of this study is to develop formulations aimed to enhance both the therapeutic potential of GapmeRs and their translation to the clinic. To achieve this objective, formulations encapsulating GapmeRs with a condensing agent within LPHNPs formulated with PLGA, DC-Chol, and DOPE-mPEG_2000_ will be developed using two different techniques: conventional bulk nanoprecipitation and microfluidic technology. The optimal formulation obtained by each technique will be identified in terms of smaller size (limit < 200 nm) and polydispersity index (PdI) (limit < 0.3) and positive ZP. Subsequently, a comparative study of EE, production yield, stability, cellular uptake, endolysosomal localization and gene silencing will be performed to determine the efficacy of the selected formulations and, at the same time, explore the production translation from a conventional bulk method to a more easily scalable microfluidic technology.

## Materials and methods

### Materials

Poly (D, L-lactide-co-glycolide) (PLGA, Resomer® RG 502, Mr MW 7,000–17,000) was provided by Evonik (Germany), soy L-α-phosphatidylcholine (lecithin) and DC-Cholesterol (3β-[N-(N’,N’-dimethilaminoethane)-carbamoyl-cholesterol hydrochloride) were obtained from Avanti Polar Lipids (USA). DOPE-mPEG_2000_ (1,2-distearoyl-sn-glycero-3-phosphoethanolamine-N-[methoxy(polyethylene glycol)-2000] (ammonium salt)) was provided by Nanosoft Polymers (USA). Chitosan hydrochloride (CHT, Protasan® UPCL-113, Mr MW: 50,000-150,000, DA: 82.5%) was purchased from Novamatrix (Norway). Protamine sulfate (Mr MW: 5,000–10,000) was purchased from Sigma-Aldrich (USA),

The specific GapmeR to silence the gene expression of Tob1 (5’-GAATTGCTGGTTAGAA-3’) and the control GapmeR without therapeutic activity labeled at 5′ with FAM (5’-FAM-AACACGTCTATACGC-3’ ) were provided by Integrated DNA Technologies (USA).

### GapmeR condensation

Three cationic products (protamine, DC-Chol, and chitosan) were chosen to evaluate their ability to form non-covalent complexes with the oligonucleotide and improve its encapsulation.

Protamine sulfate capacity to interact with GapmeR was assessed by electrophoresis as described in previous works of the research group [13]. Total condensation of the GapmeR occurred at a GapmeR: protamine mass ratio of 1:1. In the present study, to provide an excess of positive charges, the mass ratio used for LPHNPs preparation was 1:2.5. The charge ratio was estimated considering an average molecular weight of 7.5 kDa (Mr MW: 5,000–10,000) and an isoelectric point (pI) > 10 for protamine [23, 24]. Consequently, to have the same charge ratio for GapmeR: DC-Chol or GapmeR: CHT, an estimation of the mass ratio was carried out using an average molecular weight for CHT of 100,000 (Mr MW: 50,000–150,000) and pKa 6.5 [25], and a molecular weight of DC-Chol of 537.3 and pKa 7.8 [26]. In addition, the degree of ionization of the different products in pure water (pH 7) and at pH 5.8 corresponding to air-equilibrated water [27, 28] was also taken into account.

### Development of the lipid-polymeric hybrid nanoparticles (LPHNPs), formulation and elaboration process

The LPHNPs are prepared by two different methods. One of them is a modification of the one-step nanoprecipitation method and another is the microfluidic technique.

Regardless of the production method used, the formulations developed comprise an internal aqueous phase (fixed at 25 µl) containing the non-covalent complex of condensing agent (CHT: 0.033 mg/ml; protamine: 0.083 mg/ml and DC-Chol: 0.1 mg/ml) and GapmeR (0.033 mg/ml), an organic phase (polymer solution) and an external aqueous phase. The organic phase consists of PLGA solutions in acetonitrile (1 ml) at a concentration range of 0.75–5 mg/ml. The outer aqueous phase is a 4% ethanol-aqueous solution of lipids in different proportions. All phases are filtered through 0.45 μm syringe regenerated cellulose filters.

The mass ratio of lipid/polymer used to prepare and optimize the nanoparticle formulations is in the range of 10–50%. The molar ratios of DOPE-mPEG_2000_/DC-Chol + lecithin are fixed at 1:3. Lecithin/DC-Chol molar ratios are tested at 0:3, 1:2, and 2:1. DOPE-mPEG_2000_ concentrations range from 0.011 to 0.236 mg/ml, lecithin range from 0 to 0.136 mg/ml and DC-Chol range from 0.002 to 0.16 mg/ml.

#### Bulk single-step nanoprecipitation (SSN)

Briefly, 25 µl of the condensed-GapmeR aqueous solution are added to 1 ml of polymer solution in acetonitrile and mixed promptly by vortex to achieve a homogeneous dispersion of the condensed-GapmeR. This mixture is poured into 6 ml of hydro-alcoholic solution prepared with different amounts of lecithin, DOPE-mPEG_2000_, and DC-Cholesterol. The suspension is kept for 2.5 h in an extraction hood at room temperature (RT) under magnetic stirring to allow acetonitrile evaporation.

#### Microfluidics (MF)

**Fig. 1:**
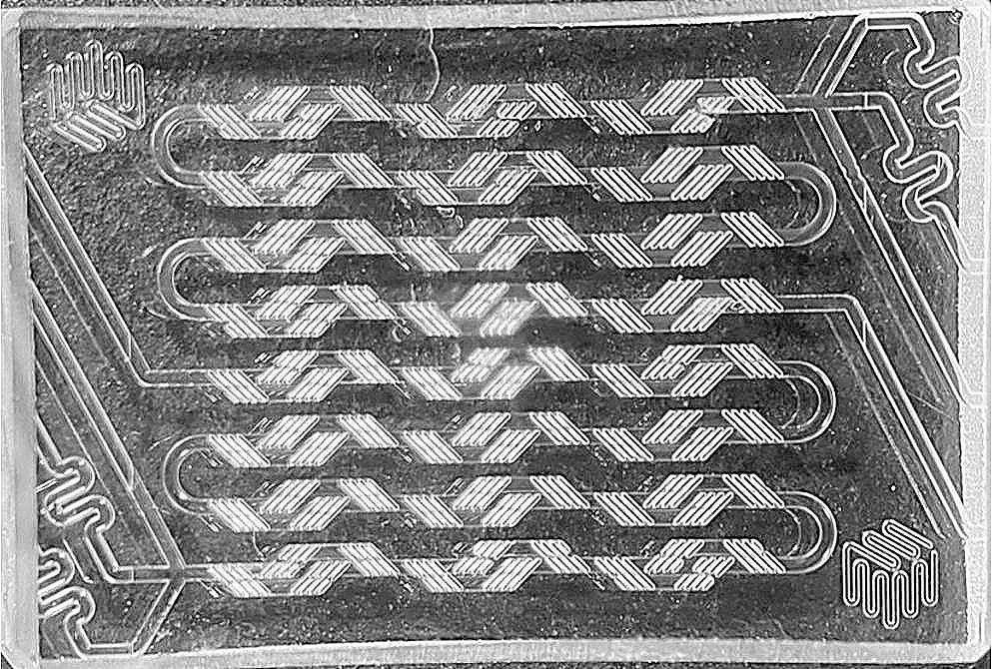
Enlarged perspective of the micromixer chip captured with stereomicroscope at 7.8 magnifications

The amounts of the lipids are dissolved in 3 ml of the hydro-alcoholic solution and loaded in 1.5 ml fractions into two syringes positioned on a syringe pump (KD Scientific, model 569, USA). This aqueous phase is pumped to the micromixer chip (Part N° 3,200,401, Dolomite Microfluidics, UK) (Fig. 1) at a flow rate range of 0.8-3 ml/min using a syringe pump (KD Scientific, model 569, USA). At the same time, 1 ml of the organic phase containing the condensated-GapmeR is also injected at a flow rate range of 0.2-1 ml/min using an automatic syringe pump (Mitos-Duo-XS-Pump, Dolomite Microfluidics, UK). Both phases get into the microfluidic chip through a ferrule with an integrated filter (Part number 3,200,245, Dolomite Microfluidics) for a run time of 5 min. Afterwards, the resulting LPHNPs dispersion was collected in a beaker and kept under mild magnetic stirring for 2.5 h in an extraction hood for organic solvent evaporation.

For elaboration process optimization by microfluidics, additional variables like Total Flow Rate (TFR) (the sum of aqueous and organic flow rates) in the system and the aqueous to organic Flow Rate Ratio (FRR) were examined.

### LPHNPs characterization

For the characterization of the different formulations, the LPHNP suspensions were concentrated by ultrafiltration using Amicon® Ultra 100 KDa MWCO filters (Millipore, USA) at 10,000 rpm for 10 min and were resuspended to a final volume of 0.4 ml.

#### Physicochemical properties of LPHNPs

The LPHNPs were characterized in terms of average hydrodynamic diameter, polydispersity index (PdI) and zeta potential (ZP) using a Zetasizer Nano-ZS (Malvern Instruments, UK). These characteristics were determined in the freshly prepared LPHNPs suspensions and after the concentration and resuspension processes. All measurements were performed in triplicate after an appropriate Milli-Q water dilution.

#### Transmission electron microscopy analysis

Transmission electron microscopy (TEM) was utilized to examine the morphology and structure of LPHNPs. This analysis is also useful to validate the particle size previously determined via dynamic light scattering (DLS). 10 µl of each concentrated sample were deposited onto copper grids and coated with a carbon membrane. Subsequently, they were stained with a 1% (w/v) uranyl acetate solution for 2 min. Finally, the samples were examined at 120 kV using a JEOL microscope (JEM 2010, Japan) and images were captured with a Gatan Orius TM camera (USA).

#### Production yield

The LPHNP suspensions were frozen at -80 °C and subsequently freeze-dried. After this process, the content was weighed on a precision balance, and the production yield was calculated as follows:

$$\eqalign{& Production\;yield\;\left( \% \right) \cr & = {{mg\;of\;LHPNPs\;obtained\;after\;freeze - drying} \over {theoretical\;mg\;\left( {sum\;of\;all\;reagent\;weights} \right)}} \times 100 \cr} $$

#### Oligonucleotide encapsulation efficiency and release

Oligonucleotide encapsulation efficiency (EE) and release profile were evaluated using fluorescently labeled GapmeR (GapmeR-FAM). LPHNP batches were prepared with a 1 µg GapmeR loading, concentrated as described above and the filtrate containing the non-encapsulated GapmeR-FAM was measured using a plate reader at 485/528 nm. Furthermore, GapmeR release assays were carried out by incubating the concentrated LPHNPs suspension (350 µl) in 10% of fetal bovine serum (FBS, Lonza, Spain) phosphate buffers (prepared with DEPC water) to a final volume of 700 µl, at pH 7.4 and at pH 5.5 and at 37 °C. In addition, the release assay was also carried out at pH 7.4 for 2 h, followed by a medium change to pH 5.5, to simulate the different pHs in the LPHNPs distribution pathway after administration. At different times, the samples were ultrafiltered (100 kDa Amicon®Ultra filters, 10,000 rpm for 10 min), the GapmeR-FAM released into the filtrate solution was quantified as previously described [29] and LPHNPs were resuspended in fresh media. The same procedure was applied to a GapmeR-FAM solution as a control of the fluorescent signal during the release assay. The assays were carried out in triplicate.

#### LPHNPs stability assay

##### Stability during storage

The storage short-term colloidal stability of LPHNPs was tested at 4 °C in water, 0.9% NaCl, and trehalose at 1.25%, 2.5%, and 10%. The LPHNP batches were suspended in the different media (final volume of 0.4 ml) and after 24 h, LPHNPs size, PdI, and ZP were evaluated by DLS.

##### Stability in simulated biological media

For this purpose, LPHNP stability was evaluated in 10% FBS in phosphate buffer saline (PBS) at pH 7.4 and pH 5.5. Additionally, LPHNP stability in a 4.5% aqueous solution of bovine serum albumin (BSA, Sigma-Aldrich, Spain) was studied. For this, concentrated LPHNPs were diluted tenfold in the different media and kept at 37 °C under orbital shaking at 300 rpm. At different time points (0, 2, 6, 24 h), 100 µl of samples were withdrawn, and the physicochemical characteristics of LPHNPs were evaluated by DLS.

#### Freeze-drying studies

For LPHNPs freeze-drying, the trehalose was selected as cryoprotectant and tested at different proportions. Each LPHNPs batch (final volume of 3 ml) containing different proportions of trehalose (1.25, 2.5, 5, 7.5, and 10% w/v) was frozen at -80 °C. Samples were introduced in the pre-cooled chamber of freeze-drier (Christ, Gamma 1–16 LSC, Germany) at temperatures of -10 °C and − 50 °C for shelf and condenser respectively and freeze-dried during 120 h (vacuum pressure 0.006 mbar). To determine the cryoprotectant effect of the different trehalose concentrations, the freeze-dried LPHNPs were resuspended in 1 ml of MilliQ water and characterized in terms of size, PdI, and ZP.

#### Cellular viability

To evaluate cell viability, murine myoblasts (C2C12 cells, ATCC) were seeded at 5 × 10^3^ cells/well in 96 well plates. Briefly, cells were incubated with NPs suspension (2 mg/ml) in complete DMEM (Dulbecco’s modified Eagle’s medium + 10% FBS, 1% Penicillin/streptomycin and 1% L-Glutamine) for 2 h. A cell group without the presence of LPHNPs, only with complete media was used as a control. After 2 h, cells were washed twice with DPBS (Dulbecco’s Buffer Phosphate, Gibco, Thermo Fisher USA) and an XTT (Roche) assay was performed following manufacturer instructions. All tests were carried out by triplicate.

#### Observation of LPHNPs cell uptake and evaluation of the intracellular distribution by confocal fluorescence microscopy

To assess LPHNPs uptake and to evaluate their intracellular distribution, C2C12 cells were seeded on sterile round glass coverslips at a density of 3 × 10^5^ cells in 35 mm dishes and incubated overnight in complete DMEM. To clarify the intracellular distribution of the GapmeR-FAM, the acidic late endosomes and lysosomes were stained by exposure of live cells to 50 nM of LysoTracker RedDND-99 (Invitrogen, USA) for 2 h. Then, cells were washed twice with DPBS and LPHNPs loaded with GapmeR-FAM (2 mg/ml) in complete DMEM were added to each well. A solution of naked GapmeR-FAM prepared with the same amount of GapmeR encapsulated in the LPHNPs was used as a control. After 2 h of incubation, cells were washed twice with PBS and fixed with freshly prepared 3.7% paraformaldehyde (PFA) for 30 min at room temperature under mild agitation, washed extensively and mounted using ProLong Gold antifade mountant complemented with DAPI (Invitrogen, USA).

Confocal images were acquired using Leica TCS SP8 laser-scanning microscope with a 40 × lens (NA = 1.4) (Leica Microsystems, Wetzlar, Germany) and image analysis was performed in Fiji (Image J, NIH, USA). Cells from three randomly selected images were selected using the freehand selection tool to create regions of interest (ROI) and the background was subtracted. For internalization analysis, GapmeR-FAM punctae were counted manually. For colocalization analysis, BIOP Just Another Colocalization Plugin (JACoP) was used [30, 31]. Channels were thresholded in the plugin and the cellular area occupied by each channel, the overlap area occupied by both channels and the Pearson’s colocalization coefficient were recorded for each ROI [32].

#### In vitro LPHNPs gene silencing efficiency

To evaluate the in vitro efficiency of LPHNPs to deliver therapeutically active GapmeRs, we exposed cultured cells to LPHNPs loaded with a GapmeR specifically targeting *Tob1* genes and assessed the silencing efficiency by real time qPCR. For this purpose, C2C12 cells were seeded on 6-well plates at a density of 2.5 × 10^5^ cells/well and allowed to attach overnight. The medium was replaced by LPHNPs loaded with either Control or Tob1 silencing GapmeRs suspended in complete DMEM at 2 mg/ml for 2 h (equivalent GapmeR concentration 50 nM). Then, cells were washed twice with DPBS and medium was replaced by fresh complete DMEM. As positive control, cells were transfected with 50 nM of non-targeting Control or Tob1 silencing GapmeRs using DharmaFECT-1 reagent (Dharmacon, UK), following manufacturer’s instructions.

After 48 h, total RNA was isolated from cell cultures using TRIzol reagent (Invitrogen, USA) following manufacturer’s instructions. Using NanoDrop microspectrophotometer the extracted RNA concentration and purity at A260/280 was confirmed and 1 µg of total RNA was reverse transcribed to cDNA using the RevertAid H Minus First Strand cDNA synthesis kit (Thermo Scientific, USA) using random hexamers as primers. To measure the gene expression of *Tob1* and *Gapdh* mRNAs the following gene-specific SYBRGreen-based primers were used: 5´-GGCACTGGTATCCTGAAAAGCC-3´ and 5´-CCAGACACTCAAATCCTGTGGC-3´ for *Tob1* and 5´-CATCACTGCCACCCAGAAGACTG-3´ and 5´-ATGCCAGTGAGCTTCCCGTTCAG-3´ for *Gapdh*. Gene expression analysis was performed in triplicates using the PowerUp SYBR Green Master Mix (Applied Biosystems by Thermo Scientific, USA) on a real-time quantitative PCR system (CFX96; Bio-Rad, USA). Once the threshold cycle (Ct) for each well was determined, the relative expression of *Tob1* mRNA was normalized to the housekeeping gene *Gapdh* using the 2^−ΔΔCt^ method [33].

### Statistical analysis

The results are presented as the mean value ± SD. SPSS software (IBM SPSS Statistics v. 26) was used to conduct a one-way ANOVA, followed by Dunnett’s post hoc test to compare the different groups with a control group or Student’s *t*-test for two samples comparison assuming unequal variance. Statistically significant differences were considered at *p* < 0.05.

## Results

### GapmeR condensation

For a 16-mer oligonucleotide, the GapmeR: protamine mass ratio of 1:2.5 can be expressed as a charge ratio of 1:2.3, regardless of the pH of pure water (pH 7) or air-equilibrated water (pH 5.8) because protamines are very basic peptides (IP > 10 [23]). Taking into account the molecular weight and degree of ionization in pure water and in air-equilibrated water of the DC-Chol and CHT, a mass ratio of GapmeR: DC-Chol of 1:5.4 and GapmeR: CHT of 1:1 was set to provide an excess amount of condensing agent to ensure the neutralization of the negative charges of the GapmeR.

### LPHNP physicochemical and morphological characterization

More than 30 different formulations were tested by SSN and MF but discarded due to their high size (> 200 nm) and/or PDI (> 0.3) or reproducibility issues. LPHNPs elaborated with a lipid/polymer mass ratio of > 20%, and regardless of both the PLGA amount used (1.25–5 mg) and the lecithin/DC-Chol molar ratio (1:2 or 2:1), have a size < 200 nm but a PdI > 0.3 and a negative ZP. However, for a lipid/polymer mass ratio of 15% using 1.25 mg of PLGA, nanoparticles with a suitable size (< 150 nm) and PdI < 0.3 were obtained, but with high ZP inter-batch variability. Consequently, lecithin was discarded from the LPHNPs composition, which also simplifies the formulations.

**Fig. 2:**
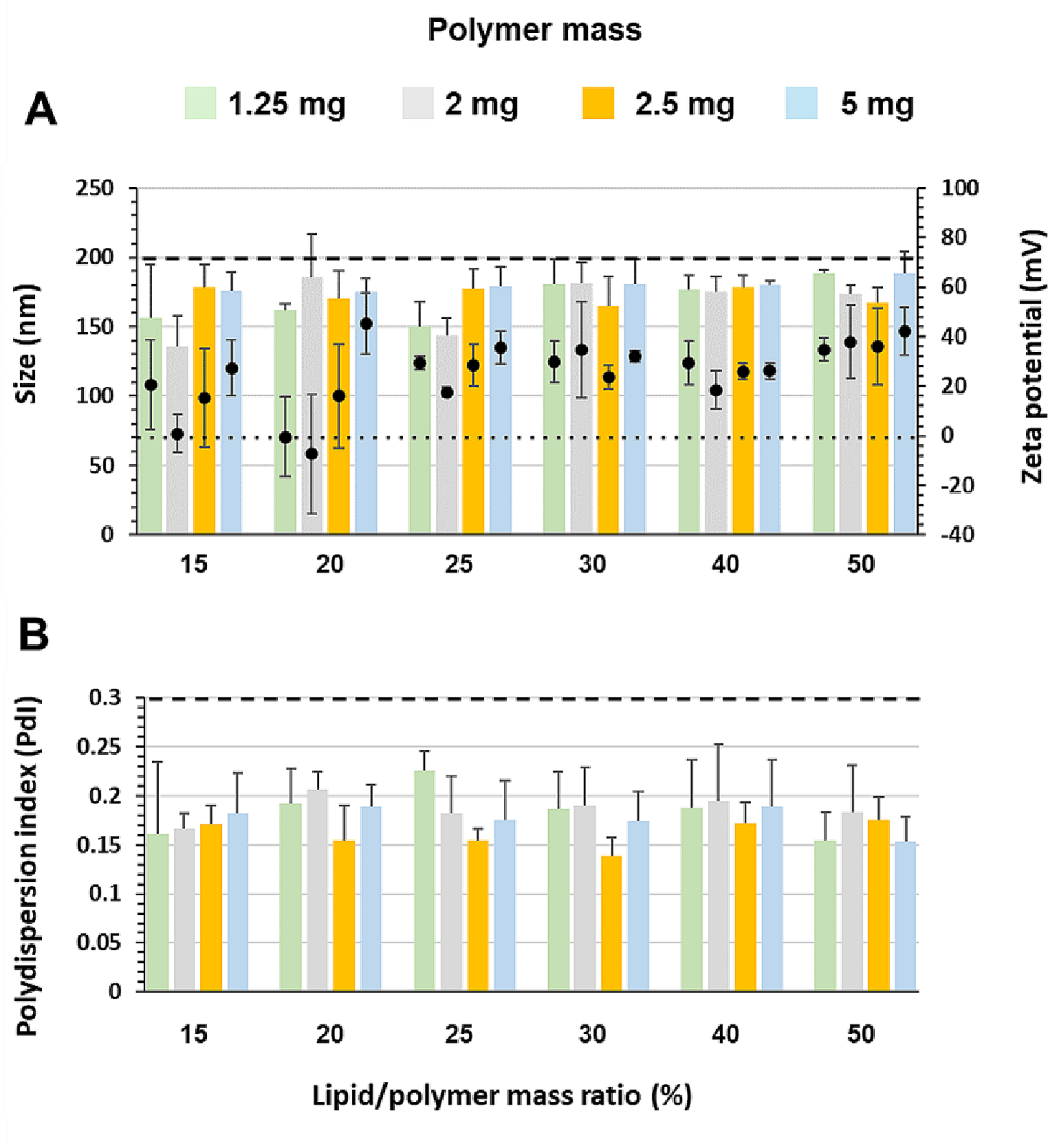
Physicochemical characteristics of LPHNPs elaborated by conventional bulk single-step nanoprecipitation (SSN) using DOPE-mPEG_2000_ and DC-Chol (molar ratio 1:3) with protamine as a complexing agent. (**A**) Average size (bars) and ZP (symbol) and (**B**) Polydispersion index (PdI). Dashed line indicated values of 200 nm and PdI = 0.3 in the respective graph and dotted line 0 mV. *n* = 3

Figure 2 shows the physicochemical characteristics of LPHNPs prepared by conventional bulk SSN without lecithin in their composition and protamine as complexing agent. The results obtained with the other complexing agents, DC-Chol or CHT, were similar (supplementary information, SI Fig. 1). The results, summarized in Table 1, show that LPHNPs made with 2.5–5 mg/ml PLGA are bigger than the ones with 1.25–2 mg/ml. This increase in LPHNPs size with polymer concentration was also described by other authors [34]. Regarding the lipid/polymer ratio, Table 1 shows that a ratio ≤ 20% produces adequate size (140–180 nm) and PdI values (0.2–0.25) but variable ZP. To get a positive surface charge that could enhance cellular uptake and promote stability by electrostatic repulsion [35], the lipid/polymer ratio has to be increased to the 25–50% range. However, to avoid excess lipids that may not bind properly to the polymeric core surface [7], the lipid/polymer ratio was fixed at 25%. Considering these results, the LPHNPs with 1.25 mg of PLGA and a lipid/polymer mass ratio of 25% (size 149.9 ± 18.07 nm, PdI 0.23 ± 0.02, ZP 29.34 ± 2.44 mV) were employed to conduct further experiments.

**Table 1:** Physicochemical characteristics (average size, PdI and ZP) of LPHNPs developed by conventional bulk single-step nanoprecipitation (SSN) using DOPE-mPEG_2000_ and DC-Chol (ratio 1:3) with different complexing agents: protamine sulfate (Prot), DC-Cholesterol (DC-Chol), and chitosan (CHT)

PLGA (mg)	Lipid/polymer mass ratio (%)	Complexant		
		Prot	DC-Chol	CHT
1.25-2	≤ 20	Size 140–180 nm, PdI < 0.25 and variable ZP		
	25–50	Size 140–170 nm, PdI < 0.25 and positive ZP		
2.5-5	≤ 20	Size 170–180 nm, PdI ≤ 0.2 and neutral or positive ZP		
	25–50	Size 170–180 nm, PdI ≤ 0.25 and positive ZP		

In order to have analogous physicochemical features with microfluidics, several parameters, such as FRR, TFR, polymer mass and complexing agent were taken into account. Although the FRR was tested in the range of 3–10, it was set to 3 because at FRR > 3 an increase in PdI was observed (data not shown) caused by a shortening diffusion length that could affect the nucleation rate [36].

**Fig. 3:**
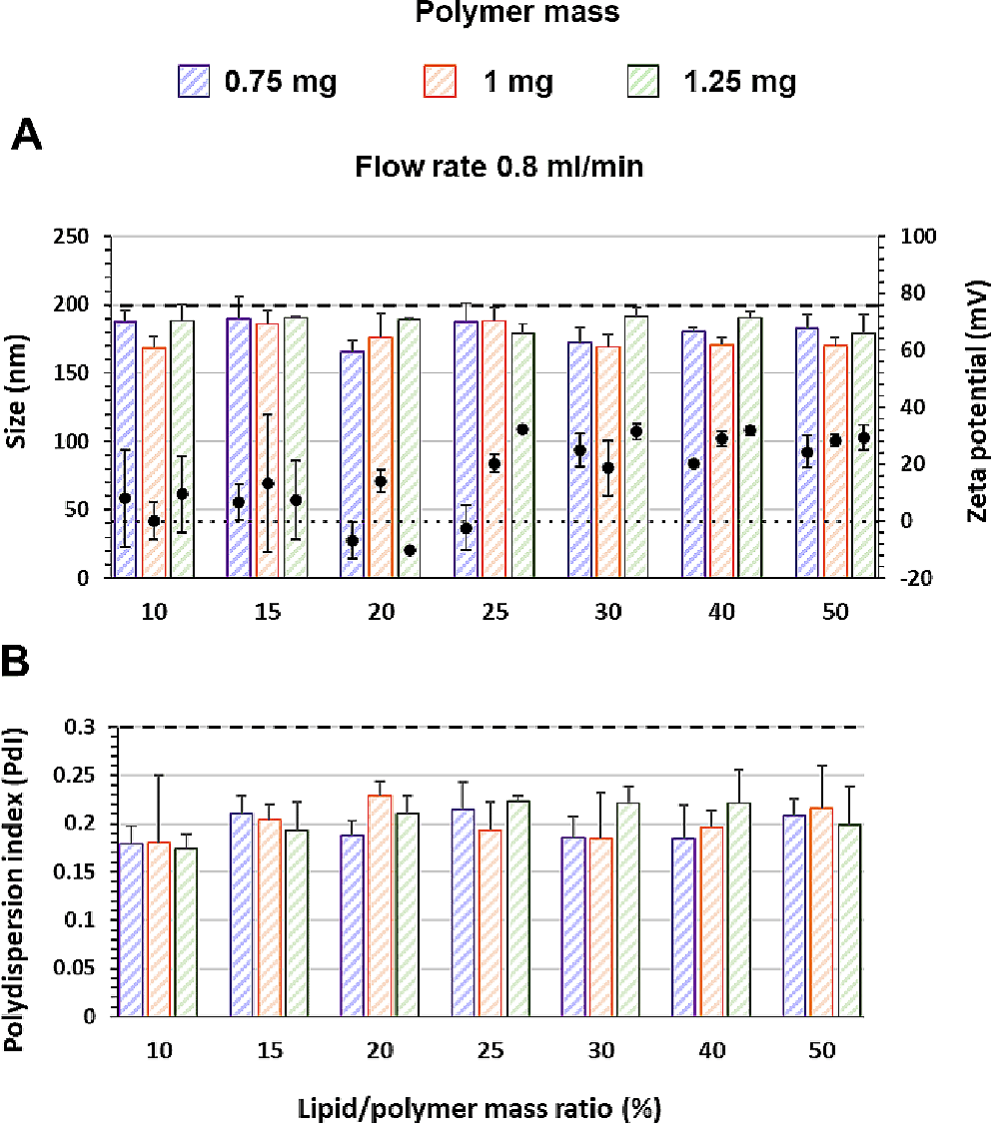
Physicochemical characteristics of LPHNPs elaborated by microfluidic (MF) using DOPE-mPEG_2000_ and DC-Chol (molar ratio 1:3) with protamine as a complexing agent. Flow Rate Ratio (FRR) of 3 and Total Flow Rate (TFR) of 0.8 mL/min. (**A**) Average size (bars) and ZP (symbol) and (**B**) Polydispersion index (PdI). Dashed line indicated values of 200 nm and PdI = 0.3 in the respective graph and dotted line 0 mV. *n* = 3

Figure 3 shows the physicochemical characteristics of LPHNPs prepared by microfluidic (MF) with protamine as complexing agent at a Total Flow Rate of 0.8 ml/min. The results obtained at the different TFR tested and with other complexing agents, DC-Chol or CHT, can be found in supplementary information (SI Figs. 2, 3 and 4). The results, summarized in Table 2, reflects that PLGA amounts higher than 1.25 mg and/or TFR levels higher than 1.5 ml/min produce chip clogging by agglomeration. This could be due to the increase in organic phase viscosity which reduces organic solvent diffusion to the aqueous phase, but also to the intricate geometries of the chips that generate pressure resistance impacting the performance of the device [37]. As a result, all the formulations tested with TFR > 1 ml/min and PLGA mass of 1.25 mg have a size > 200 nm and PdI > 0.3. LPHNPs formulated with TFR 1–1.5 ml/min do not meet the aforementioned requirements. However, the reduction in TFR down to 0.8 ml/min and the increase in lipid/polymer ratio up to 25% allowed production of positively charged LPHNPs (ZP 32.25 ± 1.36 mV), size of 179.8 ± 6.3 and a PdI of 0.24 ± 0.01.

**Table 2:** Physicochemical characteristics (average size, PdI and ZP) of LPHNPs elaborated by MF using DOPE-mPEG_2000_ and DC-Chol (ratio 1:3) with different complexing agents: protamine sulfate (Prot), DC-Cholesterol (DC-Chol), and chitosan (CHT)

PLGA (mg)	Lipid/polymer mass ratio (%)	TFR (ml/min)	Complexant				
			Prot	DC-Chol		CHT	
0.75–1.25	≤ 20	0.8–1.5	Size 180–200 nm highly variable PdI and ZP				
		1.6–3.2	Clogging of the chip				
	25–50	0.8	Size 180–190 nm and PdI ≤ 0.25	Size 160–200 nm and PdI ≤ 0.25		Size 170–190 and PdI ≤ 0.25	
			Positive ZP > 20 mV				
		1–1.5	Size > 200 nm and PdI > 0.3	Size 190–250 nm and PdI > 0.3		Size > 200 nm and PdI > 0.3	
			Positive ZP > 20 mV				
		1.6–3.2	Clogging of the chip				
1.5–2.5	< 25–50	0.8–3.2					

Flow Rate Ratio (FRR) of 3. TFR = Total Flow Rate

**Fig. 4:**
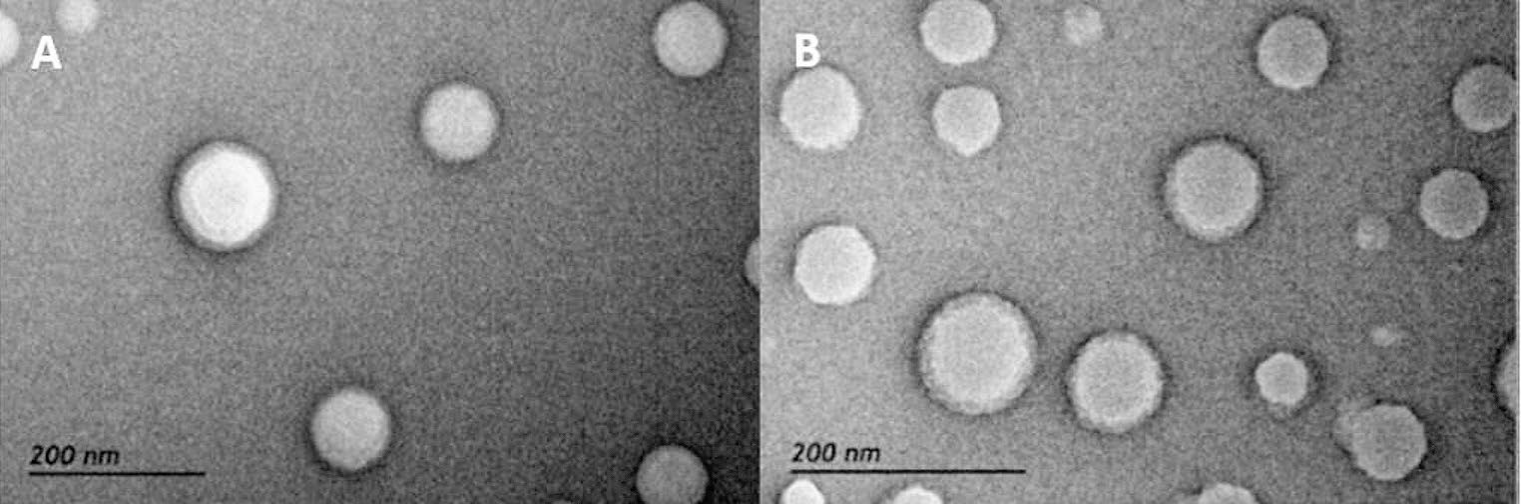
Images from LPHNPs made by (**A**) conventional single-step nanoprecipitation (SSN) and (**B**) microfluidics (MF)

Figure 4A and B present the images from LPHNPs produced by SSN and MF, respectively. Analysis of Fig. 4A confirms the presence of a homogeneous distribution in which the LPHNPs appear spherical throughout the sample. The pictures confirm the structure of the LPHNPs, formed by a polymeric core of PLGA surrounded by a lipid coating, whose components seem strongly interconnected with each other. LPHNPs obtained by MF (Fig. 4B) are as uniform as the ones made by SSN with quite similar morphological characteristics.

### Production yield, encapsulation efficiency and release of GapmeR

The production yield assessed by freeze-drying was 78% ± 3% in the case of SSN, while in MF yield increased to 86% ± 5%. These results show that both processes allow adequate production in relation to the mass of the reagents used.

Regarding the encapsulation efficiency, the values obtained for LPHNPs elaborated by SSN and MF technique with protamine were 90% ± 4% and 91% ± 7%, respectively. The encapsulation efficiency using DC-Chol (SSN EE 46.9% ± 7.4 and MF EE 45.6% ± 3.26) or CHT (SSN EE 54.3% ± 5.5 and MF EE 58.1% ± 1.3) was much lower than using protamine. In view of this finding, protamine was chosen as the condensing agent to carry out this study.

**Fig. 5:**
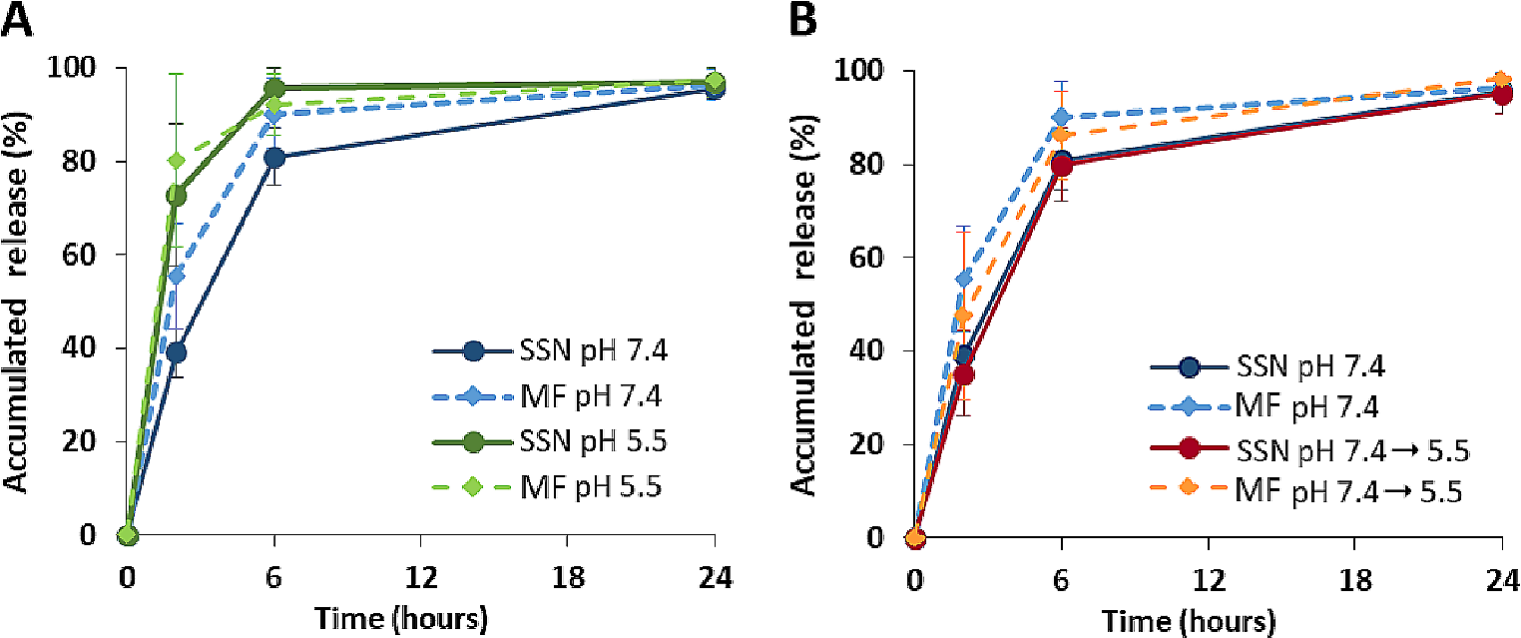
GapmeR release profile from LPHNPs (**A**) at different pHs, 7.4 and 5.5 in 10% FBS (**B**) at pH = 7.4 for the first two hours and continued at pH = 5.5 until the end of the assay. Continuous line and dotted line represent LPHNPs developed by SSN and MF, respectively. 37˚C and 300 rpm, *n* = 3

Concerning the GapmeR release from GapmeR: protamine-loaded LPHNPs (Fig. 5A), the profiles are characterized by a high burst effect followed by a slower release rate and the total oligonucleotide dose was delivered in 24 h, regardless of pH or production technique. To simulate a biodistribution process after LPHNP administration (blood and cell environment), a release assay consisting of a 2 h incubation at pH 7.4 followed by a 22-hour period at pH 5.5, was conducted. The release profiles observed in these conditions are practically superimposed on the pH 7.4 profiles (Fig. 5B).

### Stability assays

**Fig. 6:**
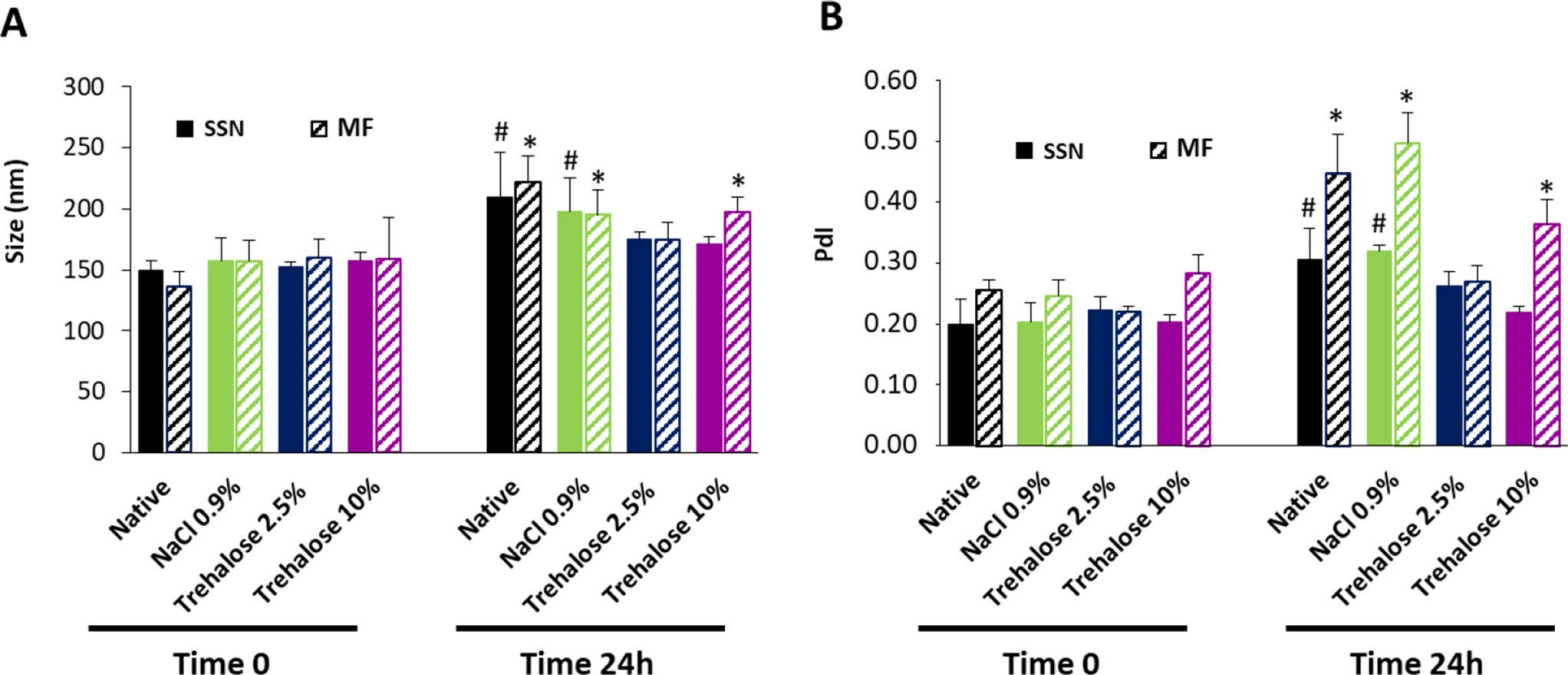
(**A**) Sizes and (**B**) PdI of LPHNPs in different stabilizers: 0.9% NaCl, 2.5% trehalose and 10% trehalose at time 0 and 24 h at 4 °C. Filled bars and striped bars represent LPHNPs developed by SSN and MF, respectively. Symbols denote statistically significant differences with native LPHNPs at time zero, prepared by SSN (#) and prepared by MF (*). *n* = 3, *p* < 0.05

Native LPHNP formulations optimized for both methods were not stable at 4 °C after 6 h (data not shown). To improve the storage of LPHNPs, they were resuspended in NaCl 0.9% to isotonize the suspension for potential future intravenous administration. However, the colloidal suspension was not stable at 4 °C for 24 h (Fig. 6). Probably the greater ionic strength imparted by the 0.9% NaCl induces the diffuse double layer of the NPs to compress with the consequent aggregation due to the attractive forces (van der Waals) becoming greater than the electrostatic repulsive ones [38]. Alternatively, two concentrations of trehalose were used: 2.5 and 10%. At high trehalose concentrations, MF LPHNPs increase their size and PdI as opposed to SSN LPHNPs. However, a low trehalose concentration maintains size and PdI for both developed formulations (Fig. 6).

LPHNPs interaction with the biological environment is a very important event to consider in the production of theragnostic nanoformulations [39, 40]. After administration, LPHNPs are immediately coated by proteins [41], but they are also exposed to pH changes. Most pH’s tissues are similar to plasma pH; however, in endosomes, pH can drop to 5.5 [42].

**Fig. 7:**
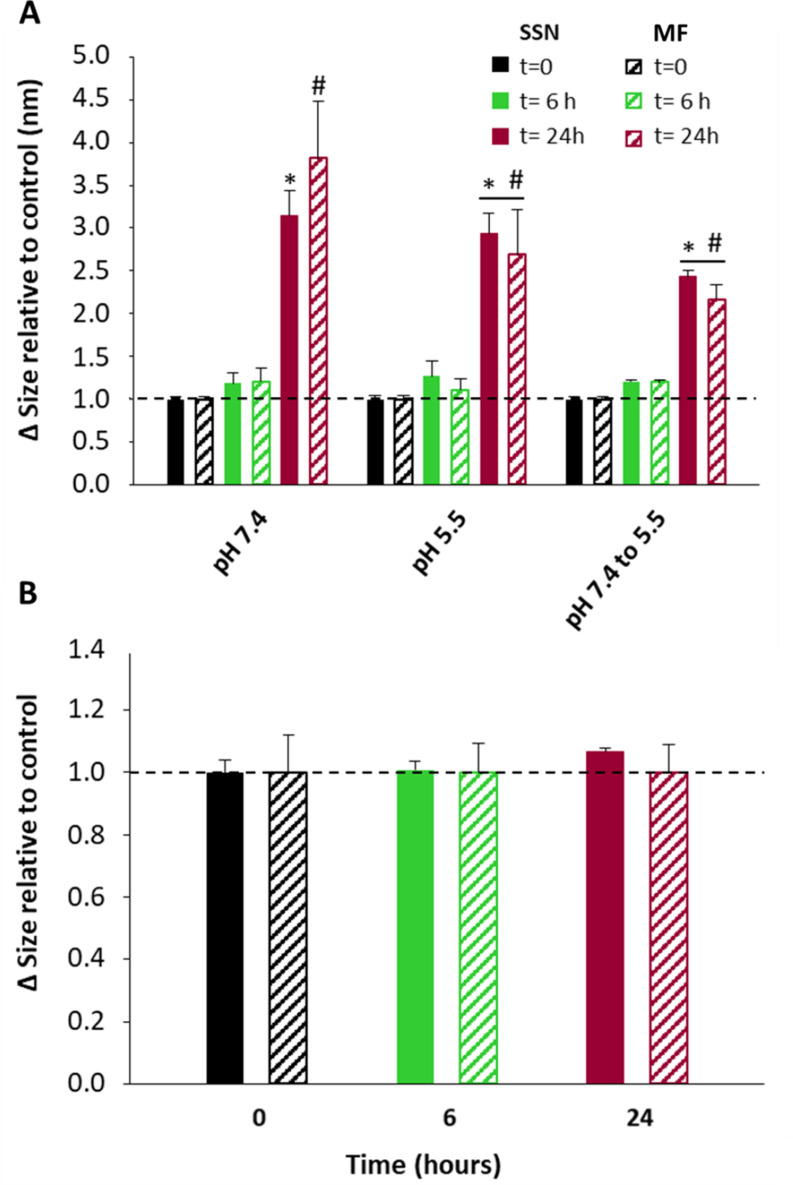
Increase in LPHNPs size elaborated by SSN and MF (**A**) in 10% FBS at different pHs, 7.4, 5.5, and 7.4 during the first 2 h and 5.5 during the rest of the assay (**B**) in 4.5% BSA at pH 7.4. Filled bars and striped bars represent LPHNPs developed by SSN and MF, respectively. Symbols denote statistically significant differences with native LPHNPs at time zero prepared by SSN (#) and prepared by MF (*). *n* = 3, *p* < 0.05

In the present work, stability of LPHNPs incubated in a phosphate buffer with 10% FBS at pH 7.4 and 5.5 was analyzed at 37 °C. Regardless of pH conditions, LPHNPs developed by both SSN and MF and incubated in 10% FBS maintained size, PdI and ZP during the first 6 h. After 24 h, the size increases 3–4 fold compared to the original value (Fig. 7A), and PdI goes up to 7 (data not shown). This effect was less pronounced in LPHNPs studied at two pHs (preincubating at pH 7.4 for 2 h).

As albumin is the major protein in serum (3.4–5.4 g/dl), LPHNP stability was also tested by incubating LPHNPs in a 4.5% BSA aqueous solution at pH 7.4. There are no significant variations in size (Fig. 7B), PdI or ZP (data not shown). Therefore, LPHNPs are stable for 24 h.

### Freeze-drying studies

**Fig. 8:**
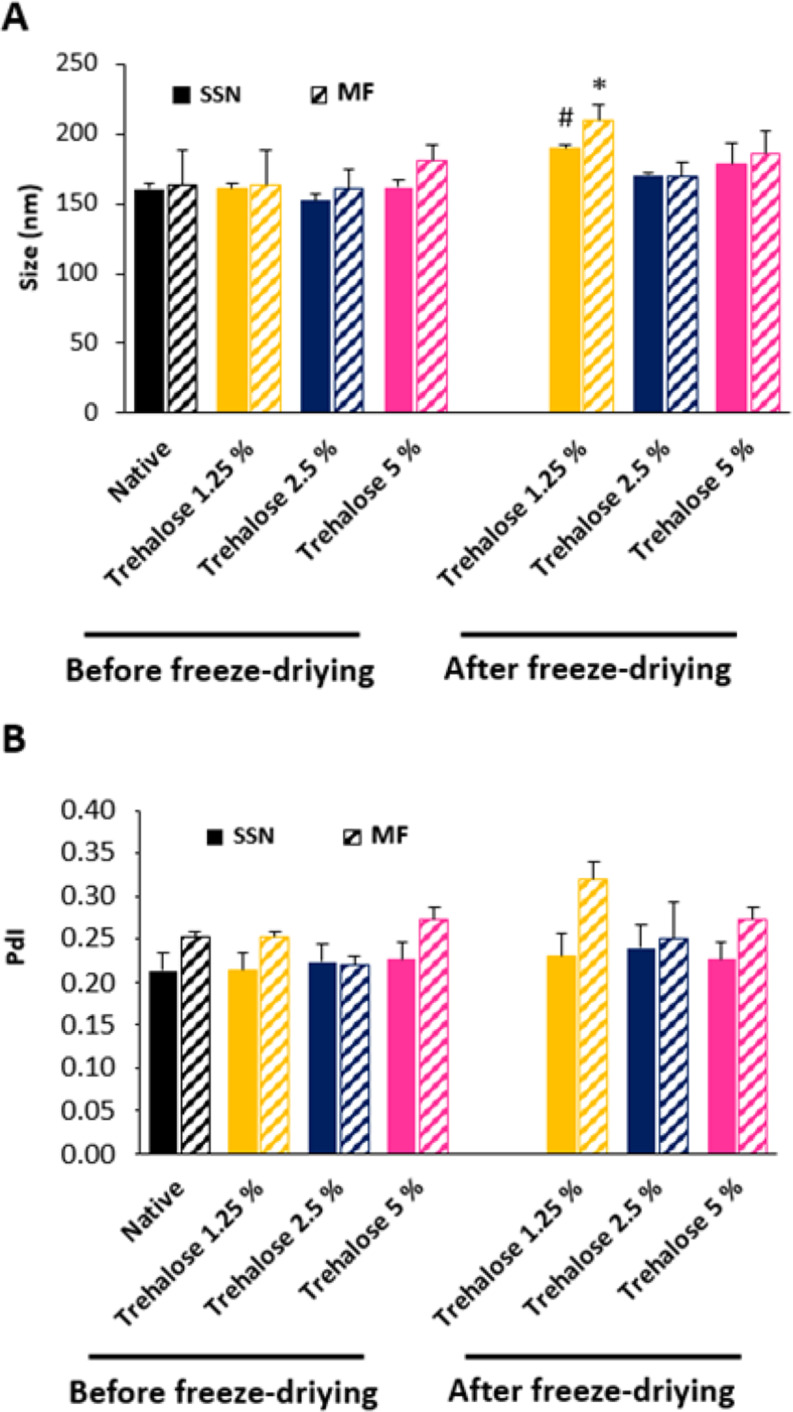
(**A**) Sizes and (**B**) PdI of LPHNPs dispersed in different concentrations of trehalose before and after being freeze-dried and resuspended in 1 ml of MilliQ water. Filled bars and striped bars represent LPHNPs developed by SSN and MF, respectively. Symbols denote statistically significant differences with native LPHNPs at time zero, prepared by SSN (#) and prepared by MF (*). *n* = 3, *p* < 0.05

The LPHNP freeze-drying was studied using trehalose in the range of 1.25–10% as cryoprotectant. After freeze-drying, LPHNPs were resuspended in 1 ml of MilliQ water by slight manual agitation and characterized in size, PdI and ZP. Highest concentrations of trehalose (7.5 and 10%) did not maintain the LPHNP characteristics after freeze-drying; sizes were greater than 1 μm and PdI > 0.9 (data not shown). This negative effect may be due to the high concentration and viscosity of the colloidal suspension which prevents the freezing of the total available water. This bound water leads to the formation of an amorphous, crystalline, or combined amorphous-crystalline phase [43]. Consequently, the lyophilization process failed and the LPHNPs were not resuspended properly. According to the results shown in Fig. 8, 1.25% of trehalose was insufficient for LPHNPs cryopreservation, while 2.5% and 5% maintained the characteristics of native LPHNPs after lyophilization, and they were easily resuspended.

### Cellular viability, cellular uptake and intracellular distribution of GapmeR

The C2C12 cell viability after 2 h of incubation with LPHNPs was around 60%, with no statistically significant differences between NPs prepared by conventional and microfluidic method (58.36 ± 6.27% for bulk SSN vs. 64.19 ± 2.31% for MF).

**Fig. 9:**
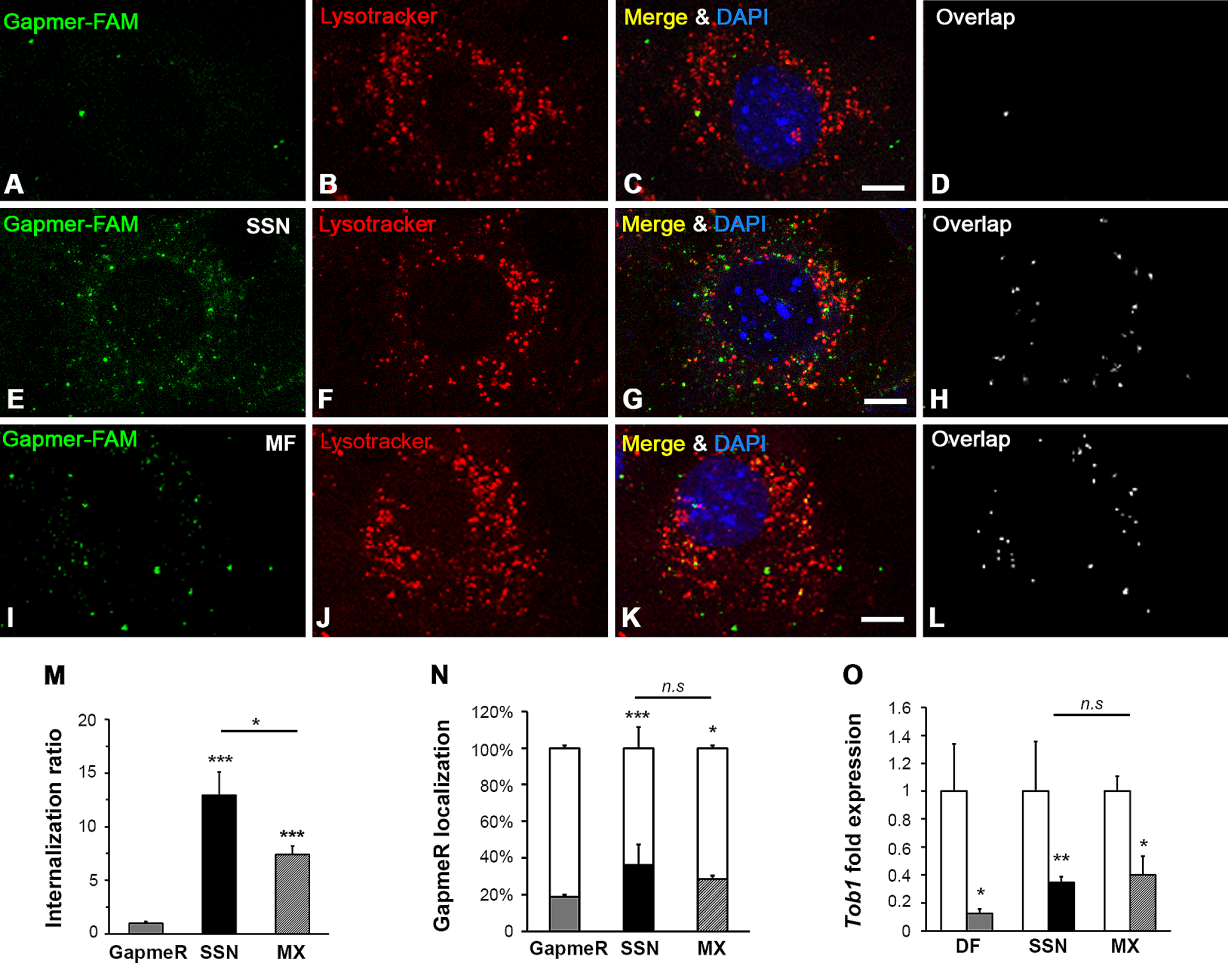
(**A**-**L**) Representative C2C12 cells exposed to naked GapmeR-FAM (**A**-**D**); (**E**-**H**) LPHNPs loaded with GapmeR-FAM elaborated by bulk SSN; (**I**-**L**) LPHNPs loaded with GapmeR-FAM elaborated by MF. Each representative confocal image is split into four sections: green channel representing FAM-GapmeR signal; red channel representing the late endosomal-lysosomal compartment labeled with the biomarker Lysotracker Red; “Merge” shows the green, red and blue (nuclei labeled with DAPI) signals; “Overlap” represents the colocalization overlays revealing as white dots those areas with GapmeR-FAM and Lysotracker Red positive signals. “Overlap” representations were obtained using the BIOP JACoP. (**M**) Quantification of the internalization ratio of GapmeR-FAM in cells exposed to LPHNPs prepared by bulk SSN or MF was calculated as the mean number of cytoplasmic FAM-positive foci per cell normalized to cells exposed to naked GapmeR-FAM (free). (**N**) Quantification using the BIOP JACoP plugin from FIJI of the percentage of GapmeR-FAM area that localizes within the endolysosomal Lysotracker-positive compartment (green-positive/red-positive “Overlap” area, colored bars) or that is distributed through the cytoplasm (green-positive/red-negative area, white bars) relativized to the total GapmeR-FAM area (total green-positive area, 100% bar). (**O**) Analysis of relative *Tob1* mRNA fold levels 48 h after treatment of C2C12 cells with either control (white bars) or anti-*Tob1* GapmeRs (colored bars) delivered by Dharmafect-1 transfection (DF) or HLPNPs elaborated by bulk SSN or MF as indicated. Scale bar: 10 μm (**A**-**L**); (*, **, ***) denotes statistically significant differences (*p* < 0.05, *p* < 0.005, *p* < 0.0005) with the naked GapmeR-FAM or among bulk SSN and MF, as indicated

To analyze the amount of cellular uptake and the intracellular distribution of GapmeR-FAM, we exposed C2C12 cells to 50 nM naked GapmeR-FAM and incubated them for 2 h at 37 °C. Naked GapmeR-FAM brightly labeled as few small cytoplasmic puncta throughout approximately 25% of the cells analyzed (Fig. 9A). In contrast, when cells were exposed to LPHNPs encapsulating GapmeR-FAM, a larger number of cytoplasmic FAM-positive puncta were observed in 100% of the cells (Fig. 9E and I). Quantification of the total number of GapmeR-FAM foci demonstrated that the internalization ratio of LPHNPs, independently of the preparation methodology, bulk SSN or MF, was significantly higher (12 and 6-fold, respectively) than the intracellular uptake of naked Gapmer-FAM (Fig. 9M).

Next, using fluorescence microscopy and confocal image colocalization analysis, we evaluated the intracellular fate of GapmeR-FAM using Lysotracker Red, a bioprobe that accumulates in the acidic environment of late endosomes and lysosomes [44]. Image analysis of C2C12 cells exposed for 2 h to 50 nM of naked GapmeR-FAM or encapsulated within LPHNPs revealed the presence of some FAM-positive punctae that colocalized with Lysotracker-positive structures revealing a partial lysosomal targeting of GapmeR-FAM in all three cases (Fig. 9A-L). Colocalization analysis showed that the percentage of GapmeR-FAM present in Lysotracker-positive endolysosomes increased after delivery with either bulk SSN or MF LPHNPs by 15% and 10% respectively when compared to naked GapmeR (Fig. 9N). It is noteworthy that the extra- and intra-lysosomal localization of GapmeR-FAM remains similar when delivered by either bulk SSN or MF LPHNPs (Fig. 9N).

Finally, to evaluate whether GapmeRs encapsulated in LPHNPs are able to escape from the endolysosomal trafficking route, we tested their pharmacological gene silencing bioactivity 48 h after the cellular uptake using anti-*Tob1* GapmeRs [45]. Quantification of *Tob1*-mRNA levels was performed after C2C12 treatment with either non-targeting Control or anti-*Tob1* GapmeRs by RT-qPCR analysis. As shown in Fig. 9O, anti-*Tob1* GapmeRs exhibit significant knockdown efficiency when compared to non-targeting Control GapmeRs (Fig. 9O), independently of the delivery methodology. As positive control, we used Dharmafect-1 to deliver GapmeRs into the cells that showed 90% of *Tob1* silencing efficiency, as previously reported [45]. The delivery of anti-*Tob1* GapmeRs by either bulk SSN or MF LPHNPs, allowed knockdown efficiencies of 60-to-70% suggesting that equivalent amounts of bioactive GapmeRs are effectively released to the cytosol to target *Tob1*-mRNAs for degradation.

## Discussion

In the present work, we take advantage of the LPHNP characteristics and the two production techniques, conventional SSN and MF, to make a platform for GapmeR delivery. Several variables were evaluated to obtain NPs with suitable physicochemical characteristics.

Regarding the lipid mixture composition, PEGylated lipids provide steric stabilization of the NP suspension during manufacturing and storage. PEGylation also prolongs circulation time and prevents uptake by the mononuclear phagocyte system [46], but has a downstream negative effect by decreasing cellular uptake. In this work, to achieve faster dePEGylation after administration, DOPE-mPEG_2000_ was chosen instead of the more widely used DSPE-PEG because of its greater dissociation rate from NP surface conferred by its unsaturated chain [47]. Similarly to cholesterol, DC-Chol strongly interacts with polymer, imparting stability to the lipid shell. The positive charge of DC-Chol improves cellular uptake and at the same time acidic pH facilitates endosomal escape [48]. Additionally, it would improve NPs stability by electrostatic repulsion during storage. The effect of lipid/polymer mass ratio was tested in the range of 10–50%. At low lipid/polymer mass ratio (≤ 20%), NPs with variable Z-potential by SSN (Table 1) and highly variable PdI and ZP or even a clogging of the chip by MF (Table 2) were produced, which indicate insufficient amount of lipids to complete core coating. The NP characteristics were not affected by the lipid/polymer ratio in the range of 25–50%. Thus, to prevent the formation of micelles or liposomes from the lipids not incorporated into the NP surface, the lipid/polymer mass ratio was set at 25%. As expected, an increase in particle size was observed when increasing PLGA concentration. Concentrations higher than 1.25 mg/mL were excessively viscous for the preparation of NPs by MF, producing chip clogging due to the low solvent diffusion rate.

Additional variables have to be considered for the NPs production by MF. TFR and FRR are the most mentioned parameters that influence the NPs size and PdI. An increase in both TFR and FRR leads to a decrease in size and PdI of lipid, polymeric and lipid-polymeric NPs. However, the micromixer design plays an important role in optimizing the process. Comparison of our results with others previously reported is difficult due to the wide variety of microchips used, which to some extent act as a limiting factor in the TFR and FRR that can be used. It has been reported that increases in FRR between 3 and 10 fold, depending on the geometry of the micromixer (homemade custom-made or commercial) and evidently the composition of the phases, lead to notable reductions in particle size [49–52]. However, the same authors also point out that the FRR has a limit above which further increases have no effect or a negative effect on size and/or PdI [49–51]. With respect to the TFR, some authors also show that the size reduction only occurs up to a certain limit beyond which the effect is reversed [53].

The micromixer chip used in the present study is a commercially available hydrophilic glass device designed to efficiently mix fluids. It combines the hydrodynamic focusing flow (HFF) at the inlet followed by 12 mixing stages consisting of a series of alternate paths with different internal cross Sect. (125 μm x 350 μm and 50 μm x 125 μm - depth x width) where a repeated fluid splitting and joining take place. HFF reduces the diffusion length of the organic solvent by compressing the central organic phase with the aqueous phase injected into the two symmetrical side channels. Like other authors [49–51], the best results were obtained with a FFR of 3 because at higher ratios (tested from 3 to 10) NPs with PdI greater than the maximum established as acceptable (0.3) were obtained. The maximum TFR tolerated by this chip is 5 ml/min to avoid internal overpressure. However, at values greater than 1.5 ml/min (tested from 0.8 to 3.2 ml/min) the chip is clogging. In the range of 0.8 to 1.5 ml/min, an increase in TFR has a negative effect, leading to NPS with greater size and PdI (Table 2), the same as the behavior mentioned by Li et al. [53].

In any case, LPHNPs with optimal size (150–180 nm), PdI (0.23–0.24) and positive Z-potential (29–32 mV), were obtained by both conventional SSN and MF. It has to be highlighted that it was possible by using the same components and in the same proportion (1.25 mg/ml PLGA, lipid/polymer mass ratio of 25% and DOPE-mPEG_2000_ at 25% molar ratio of total lipid) and setting FRR 3 and TFR of 0.8 ml/min for MF.

The TEM images show NPs with a size and PdI consistent with the results obtained by DLS and similar structure which is compatible with that of a polymeric core surrounded by a lipid shell.

One of the strategies used to improve oligonucleotides encapsulation in LPHNPs is to form, through electrostatic and hydrophobic interactions, less water-soluble larger-size neutral complexes. The three condensing agents evaluated in the present work could also improve the endosomal escape. Low molecular weight protamine is one of the most used condensing agents and with less toxicity. In fact, protamines act physiologically as condensers and DNA stabilizers in spermatozoa. However, changes in complexation efficiency have been reported between different protamines due to variations in amino acid composition and therefore in their conformation [54]. It is known that chitosan is also capable of complexing and condensing DNA molecules. The binding efficiency depends on the deacetylation degree and the presence of certain nucleotide sequences [55]. DC-Chol also forms multilaminar condensates, leaving the oligonucleotide chains confined between the lipid bilayers [56]. In our case, greater encapsulation efficiency with protamine (90%), distantly followed by chitosan (56%) and DC-Cholesterol (46%) was obtained, regardless of the preparation technique. In all cases, an excess of net charge was available for GapmeR complexation, therefore, the differences in encapsulation performances should be due to the own condensing agent structure and stability of the formed complex. According to the results, protamine sulfate was selected as a condensing agent for subsequent assays.

The stability of NPs during storage plays an important role for translating to the clinic. Despite the steric and electrostatic stabilization (ZP 29–32 mV) provided by DOPE-mPEG_2000_ and DC-Chol respectively, the NP suspensions did not maintain their physicochemical characteristics beyond 6 h. On the contrary, they were effectively stabilized using trehalose. NP suspensions in 2.5% trehalose were stable for at least 24 h at 4 °C, time enough for administration. NPs were also easily reconstituted after freeze-drying using trehalose in the range of 2.5-5% as cryoprotectant. Trehalose has also been recommended as a cryoprotectant for nanostructured solid lipid nanoparticles at concentrations in the range of 3.75–12.5% [57] and at 1–10% for polymeric particles [58]. However, in this study, concentrations higher than 7.5% do not work. Unfortunately, we have not found previous references to lyophilization of lipid-polymeric nanoparticles similar to those prepared by us. Consequently, the discrepancies with the above authors could be related with the different NP structure and composition but also to the NPs concentration to be freeze-dried.

The physicochemical properties of nanoparticles, size, shape, surface charge and surface chemistry influence the efficiency of cellular uptake [59, 60]. Upon contact with biological fluids or the culture media where they are going to be tested, NPs can change their physicochemical characteristics, which will affect their circulation time, distribution, release profile, interaction with target cells and endosomal escape. These changes are mainly due to the protein corona formation but also to the different pHs during intracellular trafficking. NPs with small sizes (30–50 nm) have greater cell penetration capacity and greater ability to escape from the mononuclear phagocyte system [61] and positive surface charges enhance the interaction with cells [62]. The usual mechanism of cellular uptake of NPs is by endocytosis. According to several authors NPs with a size lower than 200 nm preferentially use the clathrin pathway [63, 64]. Endocytic vesicles fuse with the early endosomes (pH 6–7) which mature to late endosomes (pH 5.5-6) and finally to lysosomes (pH 4.5-5). Additionally, NPs can be exocyted through recycling and exosomal exocytosis systems in any of these trafficking phases [65]. To exert their therapeutic action, oligonucleotides or oligonucleotide-NPs have to escape from the endosomal system and be released intact in the cytosol of the cell. Consequently, the behavior of the NPs was evaluated in PBS pH 7.4 with 4.5% BSA (Fig. 7B) and also in 10% FBS. In the last case at pH 7.4, at pH 5.5 and at pH 7.4 for 2 h followed by incubation at pH 5.5 to simulate the intracellular trafficking (Fig. 7A). The LPHNPs were stable for at least 24 h in 4.5% BSA while the stability was reduced to 6 h in 10% FBS. Regardless of the pH of the medium, after 24 h, the NPs increased in size, a sign of aggregation. NP aggregation could be produced by the exchange and/or the removal of the lipidic shell and by the adsorption of proteins at the NP surface. Due to the ionizable cationic DC-Chol (pKa 7.8) in the NP shell, the initial positive Z-potential (+ 29 mV) decreases to near neutrality (1–2 mV) after incubation in PBS (pH 7.4) with either BSA or FBS. The surfactant effect of BSA would keep the LPHNPs in suspension, while the complex composition of FBS with electrolytes and other substances could facilitate the loss of the lipid shell, increasing the formation of protein corona and agglomeration of the NPs. Similar behavior, after incubation in different biological fluids, in terms of decrease in ZP and stability has been reported with PLGA NPs with a cationic polymer shell [66].

The release profile of GapmeR was also affected by the pH of the medium (Fig. 5). At pH 7.4, DC-Chol is almost neutral and protein interactions are mainly by hydrophobic and hydrogen bonding forces. However, at pH 5.5, DC-Chol is fully protonated and electrostatic interactions with proteins could partially remove the lipid shell, leading to a faster GapmeR release than at pH 7.4. However, the release was not modified when LPHNPs were previously incubated at pH 7.4 and then at pH 5.5, probably because this pH change does not affect the initial structure of the already formed protein corona.

The lack of cell viability is related to the cationic lipid presence due to cell membrane destabilization [67]. Thus, an adequate cationic lipid amount is important to keep a balance between the endosomal escape and cell toxicity produced. C2C12 cell viability with the LPHNPs elaborated in the present work (60%) was similar to that described in the literature with different cell lines and LPHNPs containing cationic lipid, like DOTAP [67, 68] or BHEM-Chol [69].

To evaluate the LPHNPs cellular uptake efficiency the same dose of naked Gapmer was taken as a reference because as it previously reported single-stranded and relatively small oligonucleotides, uncharged and/or hydrophobic at high concentration can be cell uptaken and escape endosome without the intervention of any carrier [70]. Compared with the naked GapmeR, cellular uptake was much more efficient with LPHNPs independently of the elaboration methodology, conventional SSN or MF (12 and 6-fold, respectively), featuring the LPHNPs as effective vehicles for intracellular delivery. The less cell uptake efficiency of LPHNPs prepared by MF technique compared with LPHNPs by SSN is difficult to explain since their physicochemical characteristics, stability and release profiles were similar. Even though the formulation components were the same, their disposition or location in the final formulation could not be, especially in the lipid shell. The production of NPs with a relatively complex structure like LPHNPs using such an efficient mixing system could trigger variations in the nucleation and coalescence process, which would lead to a different composition than expected. A higher PEGylation and lower amount of DC-Chol at the NPs surface could reduce the cellular uptake of the elaborated NPs. Ottonelli et al. [49] reported NPs with similar physicochemical characteristics but different compositions depending on the preparation method (MF and SSN).

The performance of the elaborated LPHNPs, as a platform for gene delivery in different gene therapies, was tested using anti-Tob1 GapmeR as a model. The anti-Tob1 GapmeR activity to downregulate *Tob1* gene expression in C2C12 has been efficiently validated [45].

Our results revealed a high efficiency of gene-silencing (60–70%) determined 48 h after exposure to LPHNPs, with no differences due to the preparation methodology used, conventional SSN or MF. This evidence shows the potential of the developed NPs as delivery systems for gene therapy. Additionally, endolysosome colocalization at 2 h after cell exposure to LPHNPs, were also similar (30–40%) with both conventional SSN and MF LPHNPs.

However, the above results do not correlate to the 2-fold higher LPHNPs internalization ratio achieved by conventional SSN compared to MF. Thus, the bioavailability of active GapmeR released to the cytosol is similar using both LPHNPs, despite their cellular internalization differences. It must be taken into account that particle composition can alter both the amount of vesicle formation and the uptake mechanism, shifting from endocytosis to fusion [71]. Thus, a possible explanation is that the chemical properties of MF LPHNPs might have also activated alternative internalization processes such as the direct fusion with the cellular membrane releasing a percentage of the GapmeR directly to the cytosol. Another possible explanation is that conventional SSN LPHNPs interact more efficiently not only with cellular membrane but also with intracellular components of the early endosomal compartment, leading to longer retention times that could delay the GapmeR released to the cytosol, but also increase the probability to undergo a cellular clearance by exocytic recycling reducing GapmeR bioavailability.

## Conclusion

We have described the production of LPHNPs with PLGA, DC-Chol and DOPE-mPEG2000 by SSN and MF. The physicochemical properties were found to be sensitive to composition, polymer concentration, lipid/polymer ratio and MF parameters such as TFR and FFR, while the EE was affected by the complexing agent, the protamine giving highest EE. LPHNPs have good stability in simulated biological conditions and allow reconstitution after freeze-drying with trehalose. Furthermore, LPHNPs enhance the GapmeR cellular uptake. LPHNPs produce an efficient gene-silencing (60–70%), independently of their elaboration technique. These findings evidence the utility of the developed LPHNPs as highly effective oligonucleotide delivery systems. Furthermore, relevant aspects to consider for LPHNP production translation from a conventional bulk method to a more easily scalable microfluidic technology have been found.

## Electronic supplementary material

Below is the link to the electronic supplementary material.

Supplementary Material

## Data Availability

The datasets generated during the current study are available from the corresponding author on reasonable request.
