# Supplementary material for: Conventional and microfluidic methods: Design and optimization of lipid-polymeric hybrid nanoparticles for gene therapy
Source: Drug Deliv Transl Res. 2024 Jun 13;15(3):908–24. doi: 10.1007/s13346-024-01644-4 (PMC11782348; doi:10.1007/s13346-024-01644-4)
Supplement: Supplementary file 1 — Supplementary Material [file 13346_2024_1644_MOESM1_ESM.docx]

**Supplementary Information**


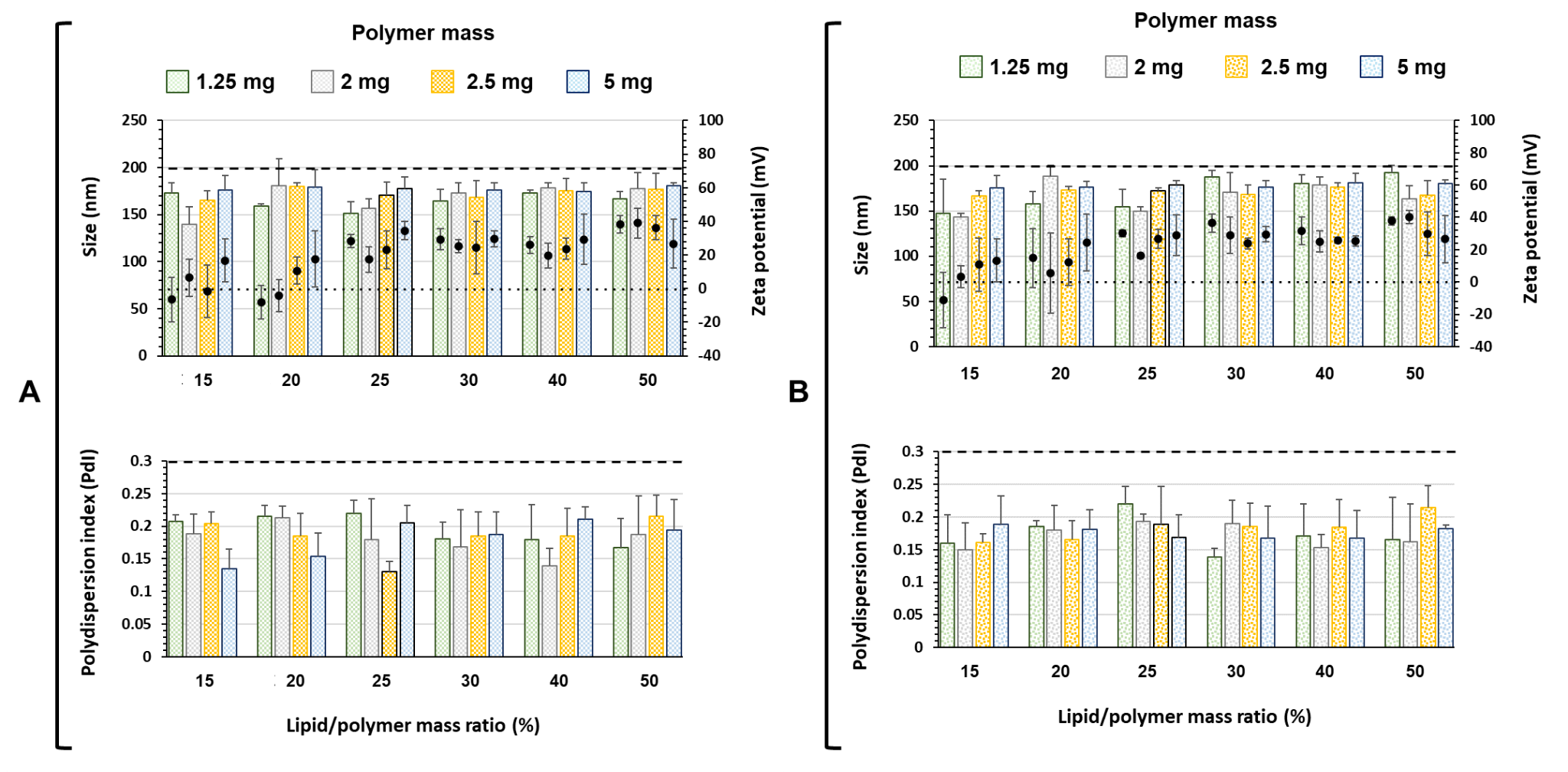


Figure 1. Physicochemical characteristics (bars (average size or PdI) and symbols (ZP)) of LPHNPs elaborated by bulk single-step nanoprecipitation (SSN) using DOPE-mPEG_2000_ and DC-Chol (molar ratio 1:3) with (A) DC-Chol or (B) CHT as complexing agent. Dashed line indicates values of 200 nm and PdI: 0.3 in the respective graphs, while the dotted line represents 0 mV. n = 3


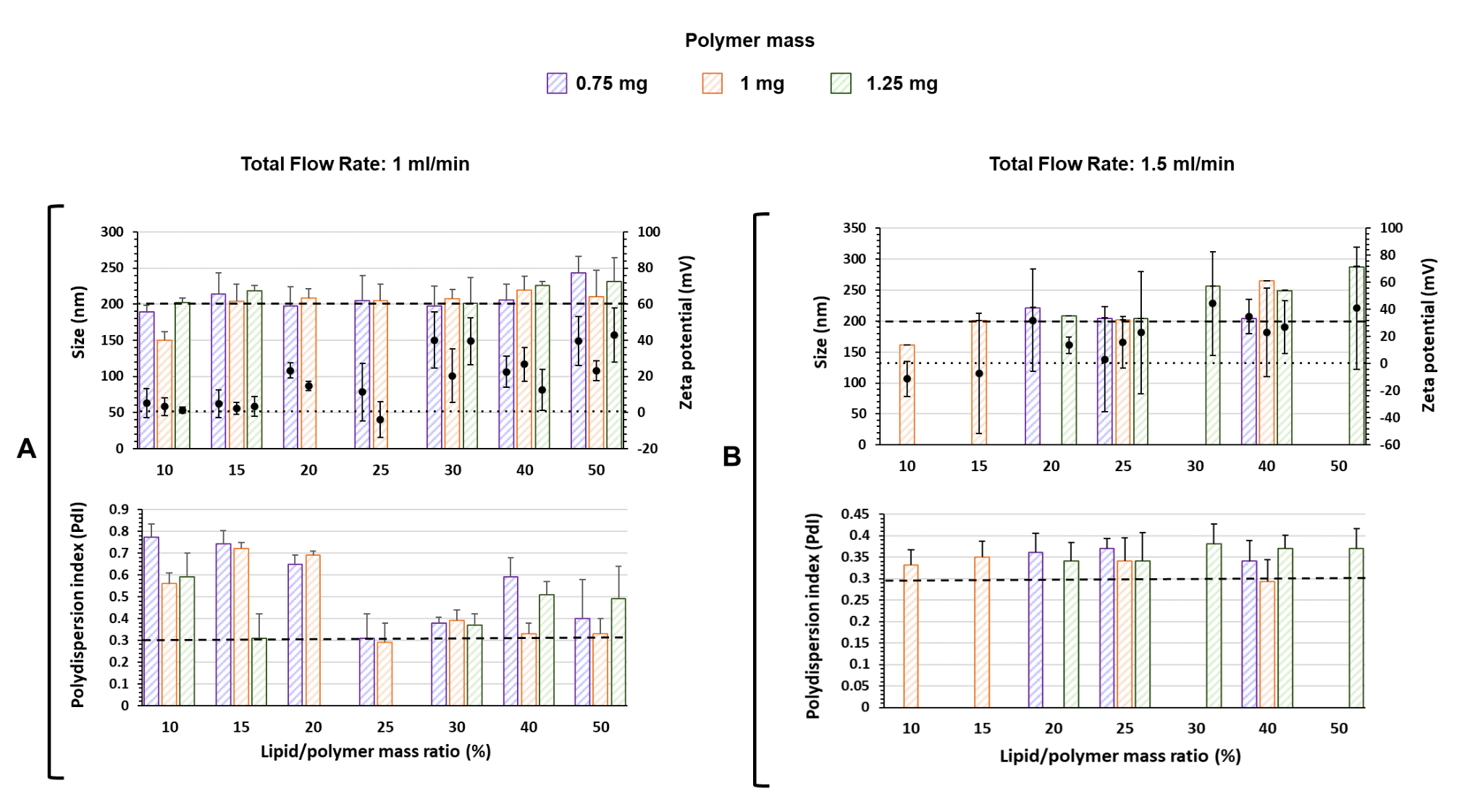


Figure 2. Physicochemical characteristics (bars (average size or PdI) and symbols (ZP)) of LPHNPs elaborated by MF using DOPE-mPEG_2000_ and DC-Chol (molar ratio 1:3) with protamine as complexing agent. Total flow rate: 1 ml/min (A) or 1.5 ml/min (B). Dashed line indicates values of 200 nm and PdI: 0.3 in the respective graphs, while the dotted line represents 0 mV. Bars not shown indicate no nanoparticle formation under those conditions. n = 3


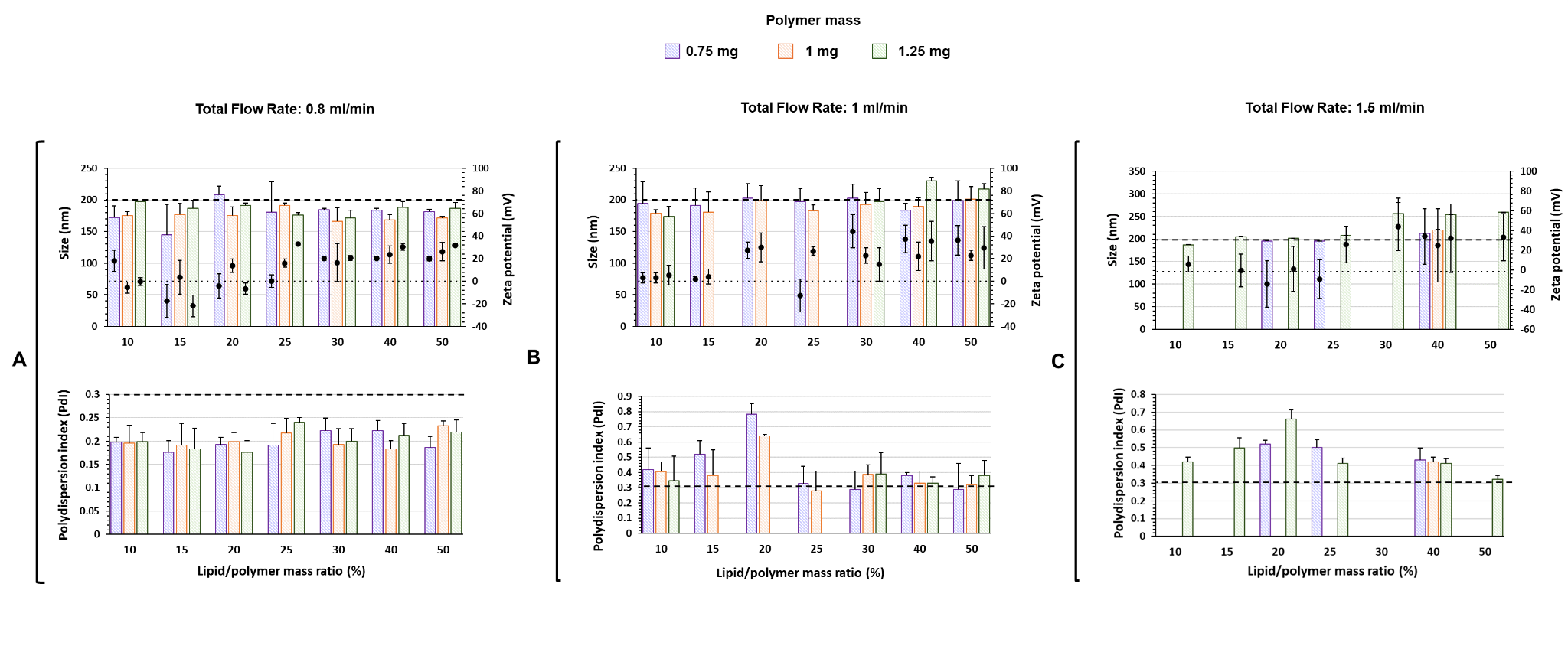


Figure 3. Physicochemical characteristics (bars (average size or PdI) and symbols (ZP)) of LPHNPs elaborated by MF using DOPE-mPEG_2000_ and DC-Chol (molar ratio 1:3) with DC-Chol as complexing agent. Total flow rate: 0.8 ml/min (A), 1 ml/min (B) or 1.5 ml/min (C). Dashed line indicates values of 200 nm and PdI: 0.3 in the respective graphs, while the dotted line represents 0 mV. Bars not shown indicate no nanoparticle formation under those conditions. n = 3


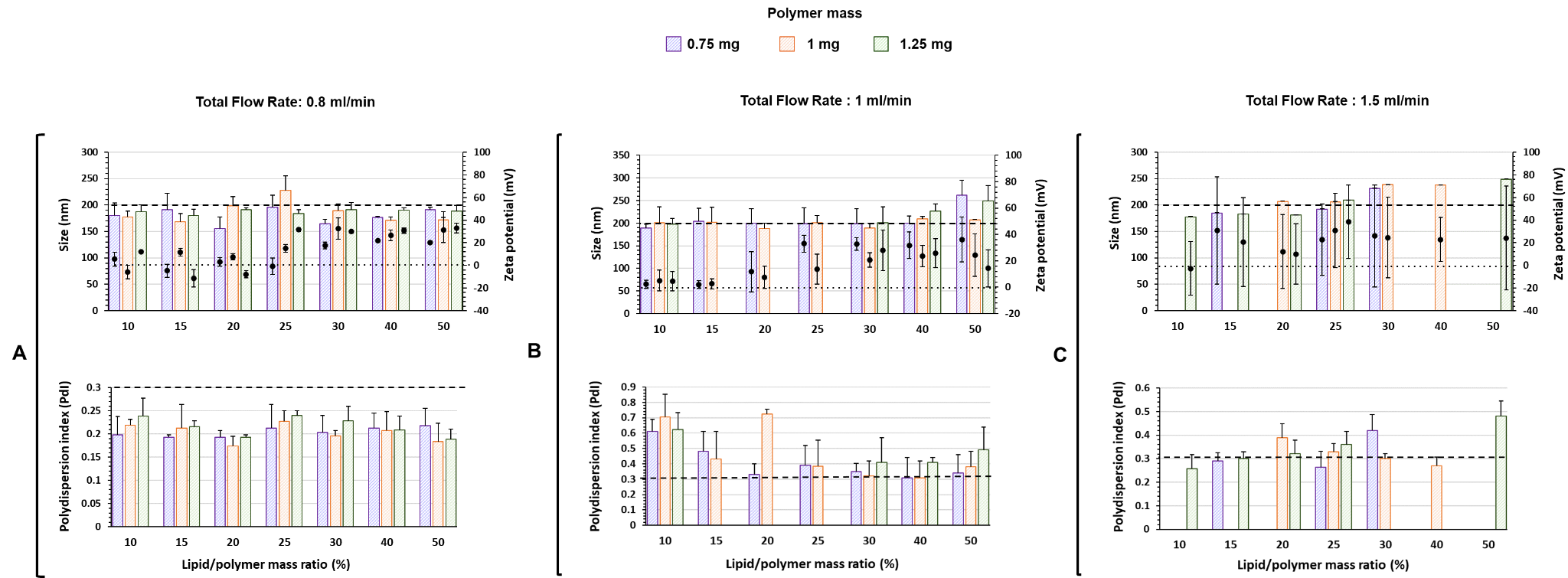


Figure 4. Physicochemical characteristics (bars (average size or PdI) and symbols (ZP)) of LPHNPs elaborated by MF using DOPE-mPEG_2000_ and DC-Chol (molar ratio 1:3) with CHT as complexing agent. Total flow rate: 0.8 ml/min (A), 1 ml/min (B) or 1.5 ml/min (C). Dashed line indicates values of 200 nm and PdI: 0.3 in the respective graphs, while the dotted line represents 0 mV. Bars not shown indicate no nanoparticle formation under those conditions. n = 3
